# Long-term results of intrathoracic chemohyperthermia (ITCH) for the treatment of pleural malignancies

**DOI:** 10.1038/sj.bjc.6601000

**Published:** 2003-06-10

**Authors:** O Monneuse, A C Beaujard, B Guibert, F N Gilly, P Mulsant, P Y Carry, M Benoit, O Glehen

**Affiliations:** 1 EA ‘Ciblage Thérapeutique en Oncologie, Université Lyon 1, Faculté Lyon Sud, 69310, Oullins, France; 2 Anesthesiologia and Intensive Care Unit, Centre Hospitalo-Universitaire Lyon Sud, 69495 Pierre Bénite, France; 3 Surgical Department, Centre Hospitalo-Universitaire Lyon Sud, 69495 Pierre Bénite, France

**Keywords:** pleural malignancies, intrapleural therapy, hyperthermia, mesothelioma

## Abstract

There is no standard treatment for patients with pleural malignancies. The aim of this prospective study was to investigate the toxicity and long-term results of a multimodality treatment consisting of surgery and intrathoracic chemohyperthermia (ITCH) for the treatment of patients with pleural malignancies. From January 1990 to August 2000, 24 patients with mesothelioma (*n*=17), fibrosarcoma (*n*=3), pleural adenocarcinoma (*n*=3) and thymoma (*n*=1) were included. The mesothelioma stages were T1 or T2 in 10 cases, and T3 or T4 in seven cases. After cytoreductive surgery, ITCH was carried out for over 60 min, at inflow temperatures less than 45°C, either with mitomycin C (*n*=7) or cisplatin (*n*=5) or both (*n*=12). One patient died from major thoracic air leaks after major decortication and pleurectomy. Seven patients had complications, one pleural clotting necessitating reoperation. After a median follow-up of 89 months, the overall 1-year and 5-year survival rates were 74 and 27%, respectively. For T1 and T2 mesothelioma patients, the median survival was 41.3 months, and for T3 and T4 tumours, it was 4.5 months (*P*=0.001). The fibrosarcoma patients are alive with no evidence of recurrence at 24, 43 and 54 months. In the conclusion, the combination of surgery with ITCH with mitomycin and/or cisplatin is relatively safe. This procedure may offer unexpected long-term survival in a selected group of patients (T1 and T2 mesothelioma patients and fibrosarcoma patients).

Malignant pleural effusion or mesothelioma has appeared increasingly in the last two decades. In France, in 1992, the frequency of mesothelioma was evaluated at 600 new cases each year (Boutin *et al*, 1997). Nevertheless, the prognosis is unfavourable with or without treatment (survival rate from 6 to 16 months). Many multimodality treatment programmes combining cytoreductive surgery, radiotherapy, chemotherapy and many experimental treatments such as photodynamic therapy, immunotherapy and gene therapy have been used (Sterman *et al*, 1999), but there is no standard treatment for these malignancies. Local treatment with antitumour drugs offers a theoretic advantage: the tumour is exposed directly to higher drug concentrations, whereas a lower incidence of toxic side effects may be expected. Combining the local effect of intrapleural chemotherapy with hyperthermia may offer an additional benefit: the effect of heat on cytotoxicity, demonstrated *in vitro* at temperatures over 42.5°C (Crile, 1963); the synergistic effect of heat and chemotherapy on certain drugs like mitomycin C (MMC) and cisplatin (CDDP) (Barlogie *et al*, 1980; Teicher *et al*, 1981). We had already reported a phase I study on the combination of surgery, intrathoracic intrapleural chemotherapy and intrapleural hyperthermia (ITCH) for diffusive malignant pleural effusions (Carry *et al*, 1993). The feasibility, the toxicity and the preliminary results of this combined management were also reported by other teams (Matsuzaki *et al*, 1995; Markman, 1997; Yellin *et al*, 2001). The objective of this prospective study was to evaluate the toxicity and long-term results of this multimodality therapy, which was given simultaneously to patients with various pleural malignancies.

## PATIENTS AND METHODS

### Protocol

The inclusion criteria were: (a) patients with diffusive malignant pleural disease; (b) no evidence of spread beyond the involved hemithorax or patients without extrathoracic metastasis; (c) cardiorespiratory function sufficient to allow the required resection; and (d) signed informed consent. The exclusion criteria were: (a) patients older than 75 years; (b) renal or myocardial failures; (c) central nervous system disease (vascular or neoplastic); and (d) WHO index >2. Malignant pleural diseases were found during thoracoscopy for preoperative cytological and histological evaluations. Patients had a complete preoperative history and physical examination, complete blood count, liver function tests, renal function tests, electrocardiogram, chest X-ray and computed tomography (CT) scan. All patients had pulmonary function tests and echocardiogram.

The procedure was realised under general anaesthesia, lateral position and one lung ventilation. A thoracotomy was performed to explore the pleural cavity, take cytological and histological samples, and for pleurectomy and/or decortication and/or wedge resection when possible. Before the surgical procedure, a Swan–Ganz catheter was inserted inside the pulmonary artery to monitor central temperature and haemodynamic parameters. A ‘pre-hyperthermia’ hypothermia (under 33°C) was realised by means of ice-bags, cold wraps on the legs (Spenco Ltd, Teynning, UK) and cooling-hat (Pinopharma, Freudenstadt, Germany to reduce the rise in systemic temperatures when the hyperthermic chemotherapy was given. The extent of surgery was tailored according to the disease and the patient's condition. Maximal attempts were made to remove all macroscopic tumour. Before closure of the thoracotomy, the equipment needed for ITCH was inserted into the pleural cavity: (a) one 30 French silicone drainage, inflow drainage (William Harvey, Bard Cardiopulmonary Division, USA), inserted at the top of the cavity; and (b) one 30 French silicone drainage, outflow drainage (William Harvey), inserted at the bottom. Thermic probes (Mallinkrodt SA and Cain SA, Lozanne, France) were also inserted: (a) in the pleural cavity; (b) one on the inflow and one on the outflow drainage (8–10 cm from the skin); and (c) one in the pericardiac space. Then thoracotomy was closed and the inflow and outflow drainages were connected to a sterile close circuit where 4 l of perfusate (Travenol Laboratory, Norfolk, England) was propelled by means of a roller pump (Cobe) at the rate of 200 cm^3^ min^−1^. The perfusate contained 0.7 mg kg^−1^ (maximum dose of 60 mg) of MMC (Kyowa, Tokyo, Japan) and/or 1 mg kg^−1^ (maximum dose of 80 mg) of CDDP. This liquid was heated through a thermic exchanger (Dideco, France) connected to a heating circuit. Intra- and extrapleural thermic probes were connected to the thermic reader (Cain SA, Lozanne, France) to be monitored each 10 min.

ITCH was carried out for over 60 min, paying close attention to respiratory and haemodynamic functions. The mean maximal inflow temperature was less than 45°C. During ITCH, blood samples were taken every 30 min to determine white cell, red cell and platelet counts, coagulation, creatinine phosphokinase (CPK), serum proteins, CRP, lactate acid and haematosis. Mitomycin C and CDDP levels in blood and perfusate were also measured. After ITCH, all the patients were placed in the intensive care unit where these differences were also determined during 1 day.

Follow-up was performed at 1 month and every 3 months in the outpatient clinic. Computed tomography scans were performed every 6 months or when a recurrence was suspected. The follow-up was updated on January 2002.

### Patients

From January 1990 to August 2000, 24 patients underwent an operation combined with ITCH with MMC and/or CDDP. From January 1990 to October 1995, ITCH was performed with MMC for mesothelioma and with CDDP for metastatic carcinoma. After the feasibility study (Carry *et al*, 1993), ITCH was performed with both for each type of tumour from November 1995 to August 2000. One patient underwent a second procedure. There were 17 males and seven females. Patients' ages ranged from 27 to 73 years (mean, 59 years). The underlying conditions were diffuse malignant mesothelioma in 17 patients, metastatic carcinoma in three patients (squamous of unknown origin in one patient and adenocarcinoma of unknown origin in two patients), fibrosarcoma in three patients and thymoma in one patient. The mesothelioma stages were T1 in five cases, T2 in five cases, T3 in six cases and T4 in one case, according to the New International Staging System (International Mesothelioma Interest Group, 1995).

Surgery consisted of decortication with pleurectomy in 11 patients, pleurectomy in seven patients, wedge resection with pleurectomy in two patients, resection of tumour with pleurectomy in three patients and explorative thoracotomy in one patient (unresectable tumour) (Table 1

**Table 1:** Patient data in chronological order

**Age (years)**	**Gender**	**Diagnosis**	**Stage**	**Surgery**	**Drugs**	**Complications**	**Site of recurrence**	**Outcome**	**Time to outcome (months)**
71	M	Mesoth	T3	Exploration	MMC		Hepatic	Dead	4
42	M	Meta Ca	M1	Pleurectomy	MMC	Air leak	Bones	Dead	74
72	M	Mesoth	T2	Pleurectomy	MMC	Bleeding	—	Dead	28
65	M	Mesoth	T2	Decortication+pleurect	MMC		Local	Dead	12
49	M	Meta Ca	M1	Pleurectomy	CDDP		Local	Dead	20
65	M	Meta Ca	M1	Wedge+pleurectomy	CDDP	Bleeding	Local	Dead	6
64	M	Mesoth	T1	Pleurectomy	MMC		Local	Dead	42
67	F	Mesoth	T1	Decortication+pleurect	MMC		—	Alive	93
62	M	Mesoth	T2	Decortication+pleurect	MMC		Local	Dead	5
45	F	Mesoth	T1	Pleurectomy	MMC		Local	Dead	41
41	M	Mesoth	T3	Pleurectomy	MMC–CDDP	Fever	—	Dead	4
66	M	Mesoth	T3	Decortication+pleurect	MMC–CDDP		Bones	Dead	5
70	M	Mesoth	T3	Pleurectomy	MMC–CDDP		Local	Dead	12
62	M	Mesoth	T1	Wedge+pleurectomy	MMC–CDDP		Local	Dead	43
62	F	Fibrosarc	T1	Resection+pleurect	MMC–CDDP		—	Alive	54
50	M	Mesoth	T2	Decortication+pleurect	MMC–CDDP		Bones	Dead	38
54	F	Thymoma	T1	Decortication+pleurect	MMC–CDDP		—	Alive	56
27	F	Fibrosarc	T1	Resection+pleurect	MMC–CDDP		—	Alive	43
62	M	Mesoth	T4	Decortication+pleurect	MMC–CDDP	Air leak	—	Dead	Postop.
67	F	Mesoth	T3	Decortication+pleurect	MMC–CDDP		Local	Dead	17
54	M	Mesoth	T3	Decortication+pleurect	MMC–CDDP	Wound sepsis	Local	Dead	16
61	F	Fibrosarc	T3	Resection+pleurect	MMC–CDDP		—	Alive	24
61	M	Mesoth	T1	Decortication+pleurect	MMC–CDDP		Local	Alive	23
73	M	Mesoth	T2	Decortication+pleurect	MMC–CDDP	Wound sepsis	Local	Alive	18

Mesoth=mesothelioma; Meta Ca=metastatic carcinoma; Fibrosarc=fibrosarcoma; pleurect=pleurectomy; MMC=mitomycin C; CDDP=cisplatin; Postop.=postoperative.

). Mediastinal lymph node sampling was taken in one mesothelioma patient and was negative. No adjuvant radiotherapy was performed. One 62-year-old patient with a T1 fibrosarcoma underwent a second procedure for local recurrence – pleurectomy and ITCH – 41 months after the first procedure. The resection margins of the initial resection were not involved.

### Statistical analysis

Data were collected and analysed on a commercially available program (Staview 4.5, Abacus Inc., Berkeley, CA, USA) and are expressed as mean, standard deviation (s.d.), median and range. The two-tailed Student's *t*-test was used to take into account differences in the mean survival of the different groups. The log-rank and Kaplan–Meier tests were used for analysis of the censored survival rates.

## RESULTS

### Thermic, biological and pharmacokinetic results

No technical problems and no thermal intolerance occurred. Neither haemodynamic instability nor respiratory instability was noticed. The maximal inflow temperature observed was 48.2°C. The mean maximal intrapleural temperature was 42.2°C (s.d. 0.7, range 40.5–43.0) and the mean maximal temperature in the pulmonary artery was 38.2°C (s.d. 0.8, range 37.0–39.1). In all patients, the temperature in the pulmonary artery dropped to normal values within 2–7 h after the end of ITCH. The blood levels of MMC and CDDP never reached the systemic toxic level (1 mg ml^−1^) on the day of ITCH and were immeasurable on the first postoperative day. There was no renal or haematological toxicity. Haemoglobin dropped by 2 points on average and total proteins by 20 g l^−1^ (s.d. 7.1). Creatinine phosphokinase increased to about 1000 IU (s.d. 366) and CRP to about 20 mg l^−1^ (s.d. 20) on the first postoperative day.

### Clinical results

The mean postoperative hospital stay was 13 days (range 9–29). The single operative mortality (hospital 30-day mortality) was related to complications secondary to major thoracic air leaks, on the 15th postoperative day, after extensive decortication and pleurectomy for the treatment of a T4 mesothelioma. Six additional patients had surgical complications: an aeric leak occurred in a 42-year-old man resulting in his hospital discharge being somewhat delayed; pleural clotting occurred in two patients necessitating surgery in one case before recovery; wound abscesses occurred in two patients without necessitating new surgery; and one patient presented a prolonged hyperthermia syndrome without bacteriological standpoint documentation.

No complication occurred after the second procedure for the patient who presented a local recurrence of fibrosarcoma. No late complications were observed.

### Survival results

The median follow-up was 89 months (range 23–149). Seven patients are still alive at 18–93 months after the surgery. There is no evidence of ipsilateral pulmonary or pleural disease in five survivors regardless of disease type. The 1-, 2-, 3- and 5-year actuarial survival rates were 74, 56, 51 and 27%, respectively. The overall median survival was 21 months. Among 16 patients who died at follow-up, the first site of disease recurrence could not be identified with certainly in most patients. In 11 patients, the disease recurred or progressed in the treated pleura and was the main cause of death in seven patients, whereas three patients succumbed to multiorgan metastases and one succumbed to contralateral pleural involvement. In three patients, bone metastasis was the first site of recurrence and patients succumbed to multiorgan metastases. All pleural recurrences were diagnosed on CT scan. Bone metastasis were diagnosed on bone scintigraphy. Two patients died with no evidence of recurrence: one from pneumonia at 4 months and one from septic shock of digestive origin. The 1-, 2-, 3- and 5-year actuarial disease-free survival rates were 64, 50, 28 and 21%, respectively. The median disease-free survival was 18 months. No significant difference was found between patients treated with MMC, CDDP or both.

**Figure 1:**
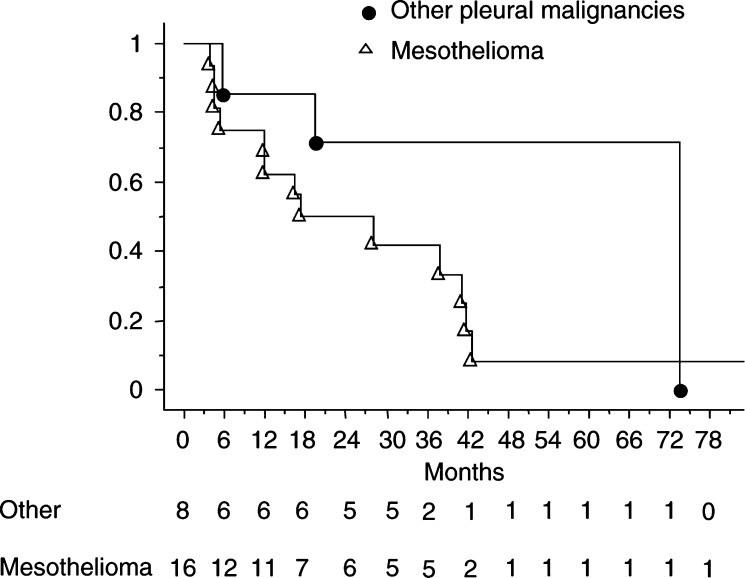
Actuarial survival (Kaplan–Meier method) of patients with mesothelioma and other pleural malignancies after surgery, intrathoracic intrapleural chemotherapy and intrapleural hyperthermia.

**Figure 2:**
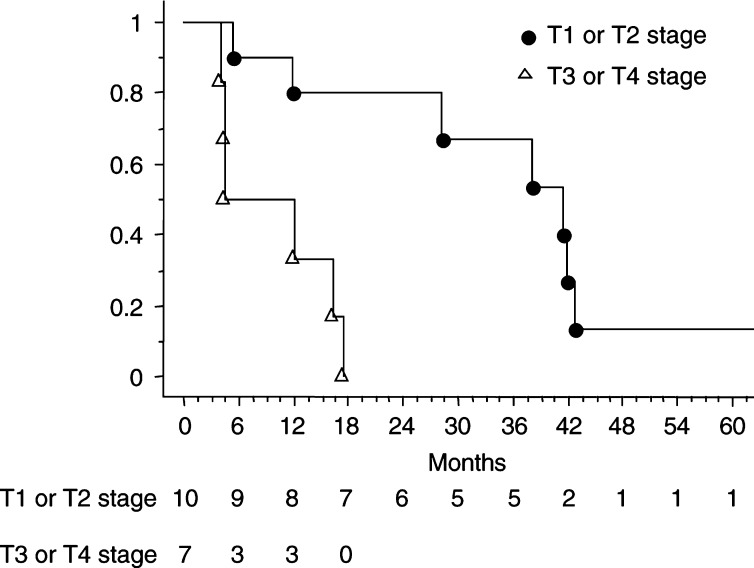
Actuarial survival (Kaplan–Meier method) of mesothelioma patients with T1 or T2 tumours and with T3 or T4 tumours after surgery, intrathoracic intrapleural chemotherapy and intrapleural hyperthermia.

In patients with mesothelioma, the 1-, 2-, 3- and 5-year actuarial survival rates were 69, 50, 42 and 8%, respectively (Figure 1

). The overall median survival was 18 months. The median survivals of mesothelioma patients with T1 or T2 stage and with T3 or T4 stage were 41.3 and 6 months, respectively (*P*=0.001). The 1- and 3-year actuarial survival rates for patients with T1 or T2 stage were 80 and 53.3%, respectively (Figure 2

). The 1-year survival rate was 50% for patients with T3 or T4 stage with no patient alive at 2 years.

In patients with other pleural malignancies, the overall median survival was 33 months. The patient with thymoma is alive with no evidence of recurrence, 56 months after the procedure. The three patients with fibrosarcoma are alive with no evidence of recurrence at 24, 43 and 54 months but after a second procedure for recurrence in one case. The three patients with metastatic carcinoma died from local recurrence in two cases (at 6 and 20 months) and from bone metastases in one case at 74 months.

## DISCUSSION

Patients with malignant pleural disease, either metastatic or primary (mesothelioma), have a poor prognosis, with an average survival of patients with pleural metastasis which is inferior to 6 months. The median survival reported for patients with mesothelioma ranges from 6 to 16 months. Multimodality approaches have been of some benefit in prolonging the survival of very highly selected subgroups of patients, but they have had a relatively small impact on the majority of patients diagnosed with this disease (Sterman *et al*, 1999). Recently, peritoneal carcinomatosis has been treated by cytoreductive surgery and intraperitoneal chemohyperthermia (IPCH), with interesting results in many specialised teams (Glehen *et al*, 2002). Radical resection or cytoreductive procedure seems to prolong survival (Sugarbaker *et al*, 1999) and locoregional chemohyperthermia offers many theorical advantages. The efficacy of hyperthermia against malignant cells has been demonstrated (Crile, 1963). On the other hand, hyperthermia has the potential to modify the cytotoxicity of anticancer drugs and to convert some other drugs to cytotoxic agents having no considerable toxicity at normal temperatures. Mitomycin C cytotoxicity, as for CDDP, was found to be enhanced by heating at 41–42°C for 1 h (Barlogie *et al*, 1980; Teicher *et al*, 1981). Moreover, local chemotherapy may have the advantage of exposing the tumour directly to higher drug concentrations and causing less toxic systemic effects. Our experience of IPCH shows that the device is safe and reliable for patients with peritoneal carcinomatosis, so we tried this method in the pleural cavity (Gilly *et al*, 1999). We choose MMC and CDDP because their antitumor effects and intraperitoneal or pleural pharmacokinetics are highly predictable and have been studied most extensively (Rusch *et al*, 1999; Matsuzaki *et al*, 1995; Hettinga *et al*, 1997; Ratto *et al*, 1999). The maximum dose of MMC and CDDP was 60 mg (0.7 mg kg^−1^) and 80 mg (1 mg kg^−1^), respectively. With these doses, the systemic drug concentrations never reached the systemic toxic level and were lower than during IPCH (Carry *et al*, 1993). This difference could be explained by the lower absorption capacity of the pleural membrane and by the enhanced local tissue concentrations after hyperthermic perfusion (Ratto *et al*, 1999). It may explain the good tolerance of this treatment. Other authors used higher doses of CDDP (80–100 mg m^−2^) for intrapleural chemohyperthermia, and they did not report renal or haematological toxicity (Yellin *et al*, 2001).

The reported study confirms that the combination of surgery and ITCH is feasible and relatively safe with mortality and morbidity rates, which did not exceed 4 and 25%. Recently, The Netherlands Cancer Institute reported mortality and morbidity rates of 0 and 47%, respectively, after treatment of 14 patients with pleural malignancies by cytoreductive surgery and ITCH with CDDP and adriamycin (De Bree *et al*, 2002). Moreover, in a previously published study of 26 patients treated with the same combined treatment (CDDP alone for ITCH), the mortality and morbidity rate did not reached 4 and 31%, respectively (Yellin *et al*, 2001). Hence, in all the studies reporting ITCH procedures, only two postoperative deaths occurred: one due to a technical problem and one due to a massive aeric leak after extended decortication and pleurectomy for a T4 mesothelioma, which was certainly not a good indication. We did not observe empyema, which mainly occurred in patients who underwent pneumonectomy (not performed in our study). Its incidence seems to be enhanced by radiation, hyperthermia and photodynamic therapy, which insult the microvascularisation of the pleura and the bronchial stump, rendering them more susceptible to infection (Sterman *et al*, 1999).

The previously published studies using the combination of cytoreductive surgery and ITCH for the treatment of pleural malignancies reported interesting results of survival and of locoregional disease control. The Netherlands Cancer Institute reported just one out of 11 local recurrence in mesothelioma patients and all thymoma patients were alive and free of disease (De Bree *et al*, 2002). Yellin *et al* (2001) reported a complete ipsilateral pleuropulmonary control in 17 out of 26 patients. The 1-, 2- and 3-year survival rates were 72, 65 and 44%, respectively (Yellin *et al*, 2001). However, it is important to note that in this study, there were only seven mesothelioma patients and 11 thymoma patients who have a completely different course as compared to mesothelioma with a better prognosis. The median follow-up of these two studies did not exceed 18 and 45 months, respectively. With a higher median follow-up, we reported a higher rate of local recurrence (13 out of 24 patients). However, the 1- and 5-year survival rates that were 74 and 27%, respectively, are interesting as the overall median survival (21 months).

The survival results for T1 or T2 mesothelioma tumours were significantly greater than for T3 or T4 mesothelioma tumours and achieved a median survival of 41.3 months. This could be explained by the surgical procedure. Pleurectomy alone or the combination of decortication with pleurectomy are perhaps not sufficient for T3 or T4 tumours. The disadvantage of this procedure relates to its limited cytoreduction, especially when the tumour invades the fissures. Extrapleural pneumonectomy may be more indicated in patients with T3 or T4 tumours and is currently used as a debulking procedure in some multimodality therapy (Sterman *et al*, 1999). For mesothelioma patients, our 2- and 5-year survival rates (50 and 8% respectively) compare favourably with the survival results reported with the trimodality therapy employing extrapleural pneumonectomy followed by combination chemoradiotherapy (Sugarbaker *et al*, 1998). The combination of cytoreductive surgery with ITCH has added to some extent to the survival results, but, certainly, part of the interesting survival results might be due to our strict selection process.

Regarding patients with other malignancies, the variability and small number of patients preclude any comprehensive conclusions. Nevertheless, in this group, three patients with fibrosarcoma are alive with no evidence of recurrence at 24, 43 and 54 months. The tumours were T1 in two cases but T3 in one case. For metastatic disease, two patients died from local recurrence at 6 and 20 months, but local control was obtained in one patient who died from bone metastases at 74 months. For these diseases, local treatment is not expected to cure patients, but local control may cause symptomatic relief and occasionally prolong survival. No objective preoperative and postoperative assessment of symptoms and quality of life was carried out in this study. However, regarding the subjective assessment conducted during the follow-up, we noticed an improvement of quality of life with symptomatic relief in all our patients until recurrence.

We conclude that the combination of cytoreductive surgery (pleurectomy alone or decortication/pleurectomy) with ITCH with MMC and/or CDDP is associated with acceptable morbidity. Encouraging results have been achieved in a selected group of patients regarding survival rates (unexpected long-term survival in mesothelioma patients with T1 and T2 tumours and in fibrosarcoma patients). However, many problems remain to be solved: controlled studies are necessary to evaluate clearly the benefit of this new therapeutic approach, and standardised criteria will be necessary (extension degree of surgical resection, type and dose of anticancer drugs, effect of higher intrapleural temperature, etc.).
